# Experience and findings from surveillance peer review in Nigeria, August 2017-May 2019

**DOI:** 10.11604/pamj.supp.2023.45.2.39450

**Published:** 2023-08-24

**Authors:** Abdullahi Walla Hamisu, Sume Gerald Etapelong, Isiaka Ayodeji, Banda Richard, Braka Fiona, Saheed Gidado, Samuel Luka Abbott, Aboyowa Arayuwa Edukugho, Omotayo Bolu, Asekun Adeyelu, Kabir Yusuf Mawashi, Usman Said Adamu, Peter Nsubuga, Faisal Shuaib

**Affiliations:** 1 World Health Organization, Nigeria Country Office, Abuja, Nigeria,; 2 National Stop Transmission of Polio (NSTOP)/African Field Epidemiology Network (AFENET), Aso, Federal Capital Territory, Abuja, Nigeria,; 3 Centers for Disease Control and Prevention, Atlanta, Georgia, United States,; 4 National Primary Healthcare Development Authority, Abuja, Nigeria,; 5 Global Public Health Solutions, Atlanta, Georgia, United States

**Keywords:** Surveillance, acute flaccid paralysis, peer review, stool adequacy

## Abstract

**Introduction:**

acute flaccid paralysis (AFP) surveillance is the gold standard of the Global Polio Eradication Initiative (GPEI) for detecting cases of poliomyelitis and tracking poliovirus transmission. Nigeria’s AFP surveillance performance indicators are among the highest in countries of the World Health Organization (WHO) African Region. The primary AFP surveillance performance indicators are the rate of non-polio AFP among children and the proportion of timely, adequate specimen collection. The surveillance working group of the National Emergency Operations Centre assessed the quality of AFP surveillance data in some reportedly high-performing states.

**Methods:**

we conducted a retrospective review of AFP surveillance performance indicators in Nigeria for 2010-2019. We also reviewed data in reports from four groups of surveillance peer reviews and validation visits (conducted by in-country GPEI partners) during August 2017-May 2019 in 16 states with high primary AFP surveillance indicators; the validation visits reviewed clinical information and the dates of specimen collection and onset of paralysis with caretakers.

**Results:**

there were consistently increasing AFP surveillance primary performance indicators during 2010-2016, followed by declines during 2017-2019. From the data for 16 states with peer reviews conducted from August 2017-May 2019, overall concordance of reported and “true” (validated) AFP indicator data in peer review investigations was highly variable. True AFP concordance ranged from 58%-100%, and stool timeliness concordance ranged from 56%-95%. The most common clinical causes of reported AFP cases that were not true AFP were spastic paralysis, malaria, sickle cell disease, and malnutrition. All the states that participated in peer reviews developed surveillance improvement plans based on the gaps identified.

**Conclusion:**

Nigeria has highly sensitive AFP surveillance according to reported primary AFP performance indicators. The findings of peer reviews indicate that the AFP surveillance system needs to be strengthened and well-supervised to enhance data quality.

## Introduction

In August 2020, the African Polio Eradication Regional Certification Commission certified the World Health Organization (WHO) African Region (AFR) free of wild poliovirus (WPV) transmission, with Nigeria having been the last AFR country with circulation of endemic WPV [1,2]. However, the current challenge in AFR and elsewhere is interrupting circulating vaccine-derived poliovirus (cVDPV) transmission to reach a truly polio-free world [3,4].

Acute flaccid paralysis (AFP) surveillance is the Global Polio Eradication Initiative (GPEI) gold standard for detecting cases of poliomyelitis and tracking poliovirus transmission, as well as providing data to Certification Commissions to certify interruption of poliovirus transmission [5,6]. The two primary AFP surveillance indicators are the non-polio AFP rate among children aged <15 years and stool adequacy with respect to the timeliness of case investigation. The non-polio AFP rate assesses the sensitivity of the surveillance system based on an expected rate of background illnesses (i.e. to detect poliovirus cases should they occur), and the percent stool adequacy measures the promptness and completeness of case investigations. ‘Adequate’ stool specimens are two stool specimens of sufficient quantity for laboratory analysis, collected at least 24 hours apart, within 14 days after the onset of paralysis, and arriving in the laboratory by reverse cold chain and with proper documentation [7,8].

Nigeria´s primary AFP surveillance performance indicators are among the highest in countries of the AFR [9,10]. In 2018, for example, the country reported a non-polio AFP rate of 9.1 cases per 100 000 children aged <15 years; the WHO target is 2.0 cases per 100 000 children aged <15 years. In the same year, reported stool adequacy was 96%; the WHO target for all countries is 80% of cases. Such high surveillance performance indicators across nearly all states and most Local Government Areas (LGAs), including the security-compromised States of Adamawa, Borno, and Yobe were considered unrealistically high by experts in external review [9,10]. A surveillance review with international consultants in 2016 that included field reinfestation for a small number of AFP cases indicated several discrepancies in onset and specimen collection dates compared to originally recorded information and a few reported AFP cases not being acute and/or not associated with flaccid limb weakness. In addition, the first outbreak response assessment (OBRA) conducted in April 2017 recommended that the very high reported AFP surveillance core indicators be more carefully evaluated. These unusually high surveillance performance indicators raised concern within and outside the country and required clarity, validation, and a path forward. Quality polio surveillance is essential, especially as the country is trying to aim to achieve a polio-free status.

Surveillance peer review is the appraisal, evaluation, or assessment of surveillance systems to determine their/its effectiveness by in-country GPEI partners. The overriding intent is to improve surveillance performance. Surveillance evaluation approaches differ depending on what needs to be evaluated and the desired outcomes [11,12]. Surveillance peer reviews for states with unusually high AFP core indicators had been organized and conducted by some states [13]. The national level selected four states with high core indicators for peer review in August 2017. The results showed an average true AFP concordance of 73% and true stool adequacy (timeliness only) concordance of 81% in the four states. The target expectation for both true AFP and stool adequacy concordance was ≥80% [14]. After presenting these peer review findings, the strategic group of the National Emergency Operation Centre (NEOC) requested that similar reviews be conducted quarterly. Consequently, we evaluated the AFP surveillance core performance indicators in a total of 16 states in Nigeria with high reported performance indicators to identify gaps and develop surveillance improvement plans to ensure quality AFP case detection, investigation, and verification.

## Methods

**Study area and population:** we selected the following 16 states: Adamawa, Bayelsa, Benue, Edo, Ekiti, Enugu, the Federal Capital Territory (FCT), Gombe, Jigawa, Kebbi, Kogi, Nasarawa, Plateau, Sokoto, Taraba and Yobe whose AFP surveillance indicators were very high (i.e. non-polio AFP rate of ≥20 per 100,000 of aged < 15 years and stool adequacy of ≥95%). These states cut across the country´s six geo-political zones. We conducted four rounds of surveillance peer reviews during August 2017-May 2019.

**Description of AFP detection and reporting in the country:** a suspected AFP case usually presents or is referred to a health facility by community informants (e.g. patent medicine vendors, traditional healers, and community leaders). At the health facility, such a case is detected by clinicians and reported to the facility surveillance focal person. The facility surveillance focal person reports any detected case to the LGA Disease Surveillance and Notification Officer (DSNO) who reports the case to the WHO Cluster Coordinator or WHO LGA facilitator for verification before case investigation and collection of two stool samples. Case investigation by the LGA DSNO can proceed if verifiers are not available. For suspect AFP cases that had stool samples taken but were later found not to be true AFP cases upon investigation by a verifier, these samples would be discarded if they had not reached the laboratory. Such a ‘rejected’ suspect AFP case is not entered into the AFP database but documented in a separate LGA line list. On the other hand, if stool specimens already reached the laboratory, the suspect AFP case must be entered into the AFP database and tagged with a unique code to indicate its status of ‘not true’ AFP case under current data management guidelines that do not permit the removal of any suspect AFP case from the database once it has been entered. ‘Not true’ AFP cases are excluded when calculating AFP surveillance performance indicators.

**Description of the AFP surveillance peer review process**

The following steps were implemented to conduct the peer-review process.

**Review of AFP surveillance performance:** we used the AFP database at the WHO Country Office to conduct a review of the two core AFP surveillance performance indicators, non-polio AFP rate and stool adequacy (timeliness only) for the period of 2010-2019.

**Selection of states:** the selection of states was guided by the AFP surveillance performance analysis for the most recent 12 months. States with a non-polio AFP rate of ≥20 per 100,000 aged < 15 years and/or stool adequacy of ≥95%) were selected for the four reviews. Four states were selected in each round of the peer review exercise.

**Categorization of LGAs within the selected states:** in each selected state for the peer review, LGAs with high surveillance performance of several levels were selected: 1) non-polio AFP rate of ≥50 per 100,000 aged < 15 year and/or stool adequacy of ≥99%; 2) non-polio AFP rate of 36-49 and/or stool adequacy of 95-98%; 3) non-polio AFP rate of 20-35 and/or stool adequacy of 90-94%. This LGA categorization selected all LGAs with high reported surveillance core indicators and allowed an adequate number of AFP cases for field reinvestigations.

**Line listing of AFP cases:** for the selected LGAs, a line list of AFP cases in children aged <15 years was reproduced; from this, 100 AFP cases that were reported within 90 days prior to the review and verified by the WHO Cluster Coordinator or WHO LGA facilitator to be true AFP cases with timely specimens were selected to be visited in the field during the peer review. Selecting verified AFP cases enabled evaluation of the accuracy of the AFP verification exercise; choosing 90 days reduced possible recall bias related to dates of paralysis onset and specimen collection.

**Selection of peer-reviewers:** peer review personnel were experienced surveillance officers selected from the government institutions (National Primary Health Care Development Agency, NEOC, and National Centre for Disease Control) and partners (WHO; US Centers for Disease Control and Prevention (CDC); African Field Epidemiology Network (AFENET); and core group). These personnel underwent a 1-day training at the national level before being deployed. Reviewers were not deployed to the geo-political zones where they worked under routine conditions. Reviewers conducted advocacy and briefing sessions at state and LGA levels before they proceeded to the field.

**Peer reviewing:** the peer-review personnel visited all selected AFP cases in their residences and applied a paper-based peer-review checklist (Annex 1) developed by the surveillance working group of the NEOC for every AFP case. Reviewers were escorted during field visits by state teams and the relevant DSNOs to assist in the transmission of reviewers´ knowledge and skills to the escorting personnel. State and LGA teams were debriefed at the end of each peer review, and surveillance improvement plans were developed to address the gaps identified.

**Figure:**
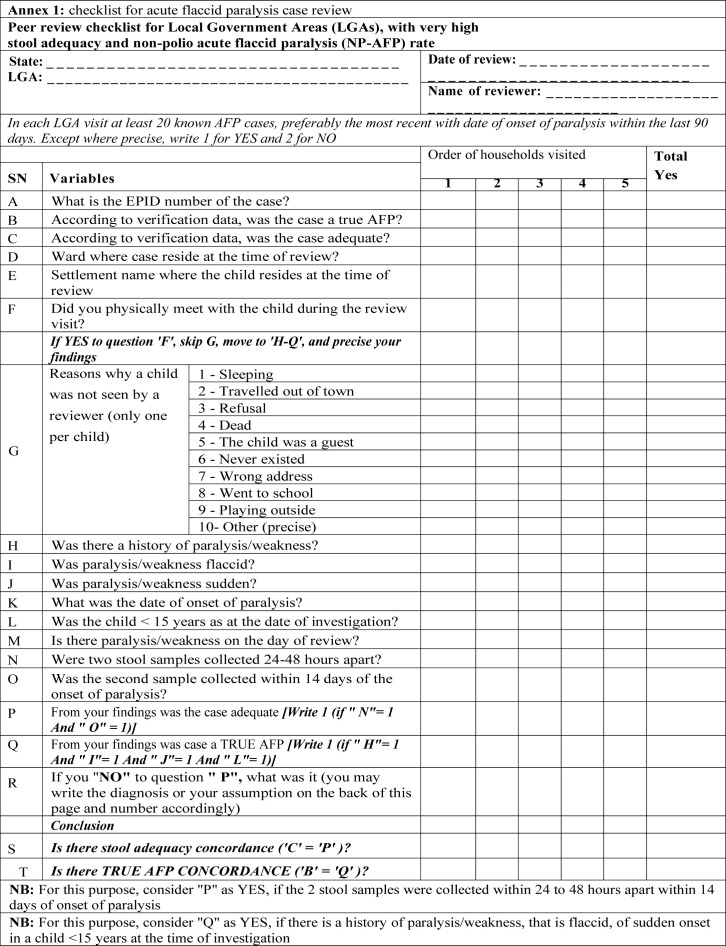


**Data collection and analysis:** surveillance peer review data were collected by visiting the residence of the selected AFP cases and eliciting responses from caregivers using a paper-based structured interviewer-administered checklist (Annex 1). Data were collated and entered into a Microsoft Excel file and analyzed. We reviewed the reports of the surveillance peer reviews to identify challenges that were encountered and appreciate surveillance gaps.

**Ethics and approvals:** permission to use the dataset used in this study was obtained from the National Emergency Operations Centre of the National Primary Health Care Development Agency. We maintained the confidentiality of the AFP patients by removing the personal identifying information from the dataset. Also, access was limited to the dataset by storing it in a password-protected computer available only to the authorized study team members.

## Results

**Figure 1:**
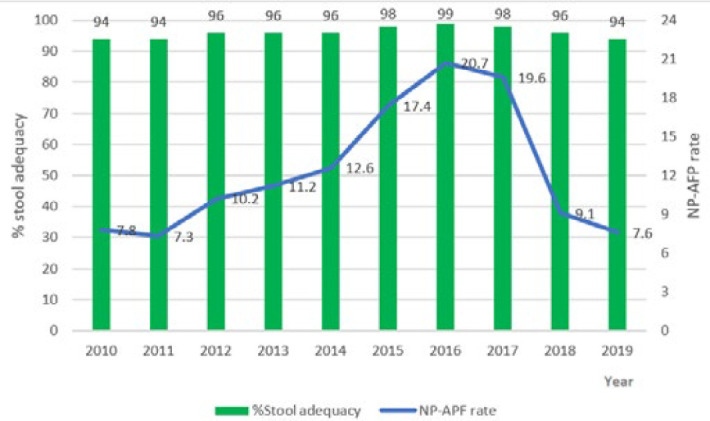
trend of non-polio acute flaccid paralysis (NP-AFP) rates and percent stool adequacy in Nigeria (2010-2019)

The review of the country AFP surveillance performance indicators prior to peer reviews showed that the reported non-polio AFP rate consistently rose from 2010 to 2016, declined slightly in 2017, and then dropped sharply in 2018 and 2019. The reported percent stool adequacy (timeliness) was consistently maintained above 90% during 2010-2019. The highest reported surveillance performance indicators of both non-polio AFP rate and stool adequacy were recorded in 2016 (Figure 1). Sub-national surveillance gaps identified during the AFP surveillance data review include the detection of orphan viruses, identification of uninvestigated missed AFP cases on facility site visits, and misclassification of polio-compatible cases [15,16].

During August 2017-May 2019, a total of 11,589 AFP cases were reported in the country. Four rounds of peer review were conducted in 16 reportedly high-performing states and the Federal Capital Territory (FCT). Among the 1,600 AFP cases selected for the peer review field reevaluation, the overall true AFP and stool adequacy (timeliness) concordance was 77% and 79%, respectively. Nine (56%) of the 16 peer-reviewed states had <80% true AFP concordance, and 8 (50%) states had <80% stool timeliness concordance (Table 1). Ekiti state had the highest concordance of true AFP (100%) and stool timeliness (95%), while Bayelsa state had the lowest concordance of true AFP (58%) and stool timeliness (56%). Most of the “not true” or false AFP cases were due to spastic paralysis, severe malaria, sickle cell disease, malnutrition, and injection abscess/cellulitis, as reported by peer reviewers (Table 2). Following debriefing meetings at state and LGA levels, surveillance improvement plans were developed to address the gaps identified (Table 3).

**Table 1:** true acute flaccid paralysis and stool adequacy concordance in peer-reviewed states (August 2017-May 2019)

Period	States	NP-AFP rate	AFP concordance	Stool adequacy	Stool adequacy concordance
**August, 2017**	Nasarawa	30.4	92%	99%	89%
	Kebbi	50.4	70%	100%	74%
	Jigawa	38.9	69%	99%	79%
	Sokoto	23.8	59%	99%	81%
**November, 2017**	Ekiti	24.3	100%	99%	95%
	Edo	26.1	92%	99%	88%
	FCT	28.3	75%	99%	68%
	Plateau	29.5	69%	99%	72%
**April, 2018**	Adamawa	20.8	85%	94%	68%
	Gombe	20.7	73%	98%	84%
	Yobe	20.7	72%	98%	69%
	Taraba	20.1	63%	98%	73%
**May, 2019**	Bayelsa	20.6	58%	98%	56%
	Benue	20.4	80%	97%	90%
	Enugu	20.5	86%	98%	84%
	Kogi	20.1	88%	96%	91%
	**National average**	**26.0**	**77%**	**98%**	**79%**

FCT: Federal capital territory; AFP: acute flaccid paralysis; NP-AFP: non-polio acute flaccid paralysis

**Table 2:** alternative diagnoses of false acute flaccid paralysis cases by peer reviewers (August 2017-May 2019)

Alternative diagnosis	Number	%
Spastic paralysis	63	16.7
Severe malaria	45	11.9
Sickle cell disease	40	10.6
Malnutrition/rickets	39	10.3
Abscess/cellulitis	30	7.9
Limb pains	19	5
Trauma	18	4.8
Cerebral palsy	18	4.8
Convulsion	11	2.9
Fracture	12	3.2
Severe dehydration/gastro-enteritis	12	3.2
Arthritis	9	2.4
Meningitis	9	2.4
Anaemia	9	2.4
Delayed developmental milestone	8	2.1
Osteomyelitis	7	1.9
Congenital abnormality	6	1.6
Snakebite	4	1.1
Central nervous system complications	3	0.8
Post measles complications	3	0.8
Pott's disease	1	0.3
Unknown	11	2.9
**Total**	377	100

**Table 3:** examples of problems identified and proposed activities in surveillance improvement plans

SN	*Problem identified	Proposed activity
1	Acute flaccid paralysis cases that are not true (no paralysis, paralysis not flaccid, weakness not sudden) are investigated and verified as true AFP	Reorientation of the Disease Surveillance and Notification Officer (DSNO) on application of the AFP case definition. Engage clinicians in major health facilities to conduct practical demonstrations during the training. Use case verification as an opportunity for capacity building.
		Enforce accountability.
		Use of appropriate interpreters, such as health workers and female guides that can enter households to get quality information from caregivers.
		Emphasis to DSNOs on quality information.
		Reorientation of the DSNOs on practical application of the AFP case definition. DSNOs should investigate AFP cases identified in health facilities with the knowledge and participation of the attending clinician.
		DSNOs should ask probing questions and also discuss with the clinicians the nature of paralysis and conduct a limited examination of the affected limb.
		DSNOs should investigate AFP cases identified in health facilities with the knowledge and participation of the attending clinicians.
2	AFP cases with inadequate stool samples taken as adequate	Reorientation of DSNOs on interview skills to elicit correct information.
		Enforce accountability.
		Orient and develop pictorial standard operating procedure (SOP) for stool specimen collection for sample collectors and caregivers.
		Reorientation of DSNOs on stool specimen collection.
		DSNOs to follow the caregiver home and collect stool specimens and geo coordinates from there. In the alternative, the case can be admitted to the facility for the purpose of stool sample collection.
		DSNOs to stop delegating responsibility for stool specimen collection to surveillance focal persons in health facilities. If delegation is necessary, surveillance focal persons should be trained.

## Discussion

The decline in the reported surveillance performance indicators observed in 2017 was a result of surveillance data quality improvement measures taken by the country. These measures were a result of external data reviews and initial peer reviews that recommended re-education of DSNOs and verifiers and reassessment of the weight of performance investigation indicators toward work performance evaluations. Surveillance peer reviews in states with unusually high AFP surveillance core indicators resulted in identifying gaps and developing surveillance improvement plans to ensure quality AFP case detection, investigation, and verification.

Overall true AFP and/or stool adequacy concordance was below 80%; reportedly high non-polio AFP rates do not necessarily imply a highly sensitive AFP surveillance. Some of the cases reinvestigated in the review did not satisfy the clinical case definition of AFP, and if proper active surveillance and supervision of field performance are weak, many true AFP cases can be missed independent of what indicators are reported [17]. Likewise, high reported stool adequacy (timeliness) may mean that the DSNOs did not have the required skills to elicit the correct date of paralysis onset, deliberately altered onset or specimen collection dates to make this indicator appear timely, or omitted the inclusion of cases reported and investigated late after onset [18]. Other surveillance peer reviews conducted within and outside Nigeria have demonstrated surveillance gaps despite high reported core performance indicators [19,20]. Standard core performance indicator analysis should be complimented with a review of all AFP surveillance performance indicators and with new techniques for AFP surveillance performance indicator analysis [21,22].

Acute flaccid paralysis is a syndrome comprising many symptoms and many conditions that bring about paralysis that may mimic polio. Only trained and experienced surveillance officers and clinicians can distinguish true AFP cases from other conditions that cause weakness or have altered gait (or use of upper limbs), especially among young children [23]. Most of the ‘not true’ AFP cases determined on reinvestigation were the result of other conditions that presented with spastic paralysis, general weakness, such as malnutrition, or local causes of limb uses, such as injection abscess/cellulitis. Injection neuritis, though a true cause of AFP, was a significant cause of AFP cases in some of these reviews. For instance, in Ekiti state, all the reported True AFP cases during the review were cases of injection neuritis [24]. This situation highlights the need for prioritizing injection safety in these states. It also raises a concern that other cases of AFP without peripheral neuropathy could have been missed.

Acute flaccid paralysis surveillance even truly meeting core performance indicators targets may not necessarily be sufficiently sensitive for low-level poliovirus transmission when paralysis may be not reported [25]; therefore supplementary Environmental Surveillance (ES) can be very important. Nigeria implements ES in all states and the Federal Capital Territory [26,27].

One of the critical limitations of this work is the risk of recall bias from caregivers concerning dates of paralysis onset during peer review reinvestigation, considering that some of the AFP cases selected were up to 90 days from notification. Also, some selected suspect AFP cases could not be visited due to insecurity in the area. In addition, the findings from this study cannot be generalized to all states with high core indicators in the country. Nonetheless, the high reported surveillance performance indicators need constant monitoring; discordances in the reported core indicators need to be addressed to ensure quality and reliable information. One of the key public health implications of our findings is that surveillance data quality improvement is possible if we insist on ‘quality’ and demystify ‘quantity’ to field surveillance personnel. We should also insist that all surveillance efforts should be documented with an emphasis on processes. Also, accountability and continuous training of surveillance personnel should be ensured [28].

**What are the implications for public health practice?**

The findings of the surveillance peer reviews provided an opportunity to develop state-specific surveillance improvement plans to enhance the quality of surveillance performance indicator data.

## Conclusion

We recommend that the developed surveillance improvement plans continue to be implemented and monitored. The work plans were made sufficiently specific to address and correct many gaps identified. We also recommend ongoing monitoring and documentation of rejected and not true AFP cases. Active surveillance and supervisory visits should be well documented and emphasized as a way of ensuring surveillance performance and intervention. Finally, surveillance field staff, including the DSNOs and partners (and ES sample collectors), should be regularly trained, given feedback, and motivated to work in this highly challenging situation.

**Disclaimer:** the findings and conclusions in this report are those of the authors and do not necessarily represent the official position of the U.S. Centers for Disease Control and Prevention.

### What is known about this topic

- The Global Polio Eradication Initiative primarily relies on certification-standard acute flaccid paralysis (AFP) surveillance performance indicators to ascertain whether or not poliovirus is circulating, assuming high data quality.

### What this study adds

- Analysis of the in-country, GPEI partner-facilitated surveillance peer review data, 2017-2019 indicated that the quality of AFP surveillance performance indicator data is suboptimal.
